# A One ppm NDIR Methane Gas Sensor with Single Frequency Filter Denoising Algorithm

**DOI:** 10.3390/s120912729

**Published:** 2012-09-18

**Authors:** Zipeng Zhu, Yuhui Xu, Binqing Jiang

**Affiliations:** 1 College of Physics and Optoelectronics, Taiyuan University of Technology, Shanxi 030024, China; 2 Key Laboratory of Advanced Transducers and Intelligent Control System (Taiyuan University of Technology), Ministry of Education, Shanxi 030024, China; 3 RAE Systems (Shanghai) Inc., No. 990 East Hui Wang Road, JiaDing District, Shanghai 201815, China; E-Mails: xuyh@raesystems.com (Y.X.); bqjiang@raesystems.com (B.J.)

**Keywords:** methane, NDIR, FFT, gas sensor

## Abstract

A non-dispersive infrared (NDIR) methane gas sensor prototype has achieved a minimum detection limit of 1 parts per million by volume (ppm). The central idea of the design of the sensor is to decrease the detection limit by increasing the signal to noise ratio (SNR) of the system. In order to decrease the noise level, a single frequency filter algorithm based on fast Fourier transform (FFT) is adopted for signal processing. Through simulation and experiment, it is found that the full width at half maximum (FWHM) of the filter narrows with the extension of sampling period and the increase of lamp modulation frequency, and at some optimum sampling period and modulation frequency, the filtered signal maintains a noise to signal ratio of below 1/10,000. The sensor prototype provides the key techniques for a hand-held methane detector that has a low cost and a high resolution. Such a detector may facilitate the detection of leakage of city natural gas pipelines buried underground, the monitoring of landfill gas, the monitoring of air quality and so on.

## 1. Introduction

Methane is the main component of coal mine gas and natural gas, and it is closely connected with the people's daily activities and life. Since methane gas is inflammable and explosive, it is important to accurately detect the concentration of methane gas. Methane detectors may be classified into two categories based on their applications. One is mainly used to alarm for the explosion of methane. Since the low explosive level (LEL) concentration of methane is 5%, detectors for this purpose usually do not require a high resolution or a very low detection limit. The other is focused on detecting very low concentrations of methane (ppm level). Applications of this type may include the detection of the leakage of natural gas pipelines buried underground, for if there is a pipeline leak, the gas concentration on the Earth surface should be greatly diluted [1]. A ppm level methane detector may also be suitable to monitor landfill gas and air quality, *etc.* [2–4].

Methane sensors are based on various detection principles, such as catalytic combustion [5], metal-oxide-semiconductor (MOS) resistance [6], NDIR absorption spectroscopy [7,8], tunable diode laser absorption spectroscopy (TDLAS) [3] *etc.* Currently, most types of commercial methane sensors are not capable to detect 1 ppm level methane gas, only the TDLAS type can and usually has a minimum detection limit of 1 ppm [9]. However, the price of the TDLAS methane detector is very high compared with the other types, which prevents it from widespread application.

The NDIR type gas sensor has the advantages of relatively low-cost, high detection accuracy, high stability, fast response time [10,11], *etc.* Lowering the detection limit of the NDIR methane sensor to a low ppm level has attracted great interest in the field in recent years [12–15]. In this paper, we propose a new design of an NDIR methane sensor with the features of a relatively long optical path and a single frequency filter denoising algorithm. The dynamic measurement results of the sensor implies that a 1 ppm detection limit has been accomplished.

## 2. Experimental Principle

### 2.1. NDIR in Practice

The NDIR method for gas concentration measurement is based on Beer-Lambert law [16]:

(1) $$I_{\text{out}}(\lambda )=I_{0}\exp(-\alpha (\lambda )CL)$$

where *I_0_* is the incident light intensity from the IR light source, *I_out_* is the intensity on the IR light detector, *α*(*λ*) is the absorption coefficient at wavelength *λ*, *C* is the gas concentration, and *L* is the absorption path length.

**Figure 1.**
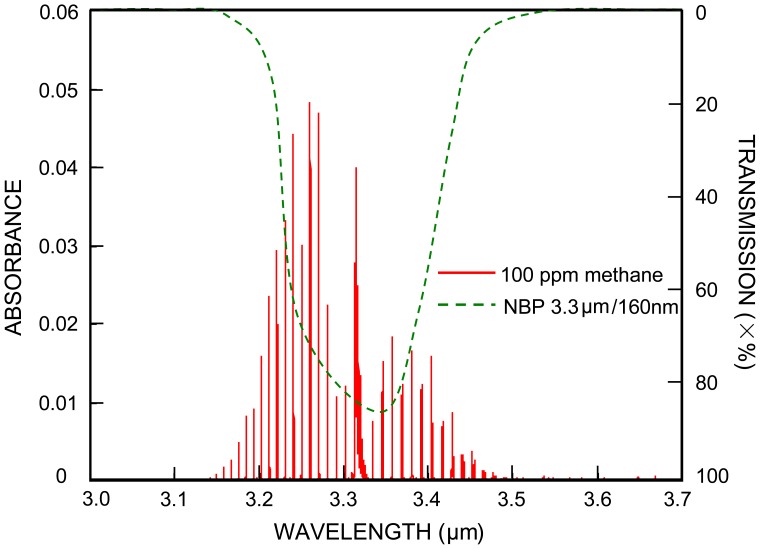
Simulation of methane absorption spectrum near 3.3 μm and transmission spectrum of the bandpass filter.

Practical consideration is necessary when Equation (1) is applied to the NDIR methane sensor case. Figure 1 shows a simulation absorption spectrum (red lines) of 100 ppm methane with a 10 cm path length, under 1 atm pressure and at room temperature. The data of the simulation spectrum were obtained from the HITRAN database [17]. Also shown in Figure 1 is a transmission spectrum of a bandpass filter (green curve), generated after InfraTec's typical filters plots [18]. The IR light detectors utilized in many NDIR methane sensors are typically integrated with a narrow bandpass filter (NPB). For example shown in Figure 1, the pyroelectric methane detector of InfraTec has a signal filter, whose transmission spectrum has a FWHM of 160 nm and is centered at 3.3 μm, around where methane has the most absorbance.

On the other hand, many NDIR methane sensors utilize a broadband light source (for example, an incandescent mini lamp) that has a continuum light emitting spectrum around 3.3 μm. Therefore the IR light passing through the NBP filter in Figure 1 is continuous whereas the methane absorption peaks are discrete in wavelength, and only the intensity of the IR light at the wavelengths that match the absorption peaks will change if there is a variation of methane gas concentration.

With the above practical consideration, Equation (1) may be modified as:

(2) $$I_{\text{out}}(\Delta \lambda )=I_{\text{non}}+I_{\text{abs}}=\sum_{\lambda ,\text{non}}I_{0}(\lambda_{\text{non}})+\sum_{\lambda ,\text{abs}}I_{0}(\lambda_{\text{abs}})\exp(-\alpha (\lambda_{\text{abs}})CL)$$

where *I_out_*(Δλ) is the total intensity over the wavelength range of the NBP filter, *I_non_* represents the total intensity from the wavelengths where methane does not absorb within Δλ, and *I_abs_* represents the total intensity at the wavelengths where methane absorbs. Moreover, the IR light source is typically driven with a square wave voltage supply, consequently the IR detector outputs a signal with a sinusoidal or triangular-like waveform, and the peak-to-peak voltage amplitude *V_p-p_* from the waveform is measured to reveal the change of gas concentration:

(3) $$V_{p-p}=kI_{\text{out}}(\Delta \lambda )=k\sum_{\lambda ,\text{non}}I_{0}(\lambda_{\text{non}})+k\sum_{\lambda ,\text{abs}}I_{0}(\lambda_{\text{abs}})\exp(-\alpha (\lambda_{\text{abs}})CL)$$

where *k* is a proportional coefficient.

For a dynamic measurement method, that is, we switch between the methane gas of a certain concentration and pure nitrogen, the difference of the *V_p-p_* is then:

(4) $$\Delta V_{p-p}=k\sum_{\lambda ,\text{abs}}I_{0}(\lambda_{\text{abs}})(1-\exp(-\alpha (\lambda_{\text{abs}})CL))$$

Finally, if noise is included, we have:

(5) $$\Delta {V^{\prime }}_{p-p}=\Delta V_{p-p}+n=k\sum_{\lambda ,\text{abs}}I_{0}(\lambda_{\text{abs}})(1-\exp(-\alpha (\lambda_{\text{abs}})CL))+n$$

where Δ*V*′*_p-p_* represents the peak-to-peak amplitude from a practical measurement, and *n* represents the voltage amplitude of the noises from all sources.

It can be seen from Equation (5) that, in order to lower the detection limit, or in other words, to enhance the SNR, we need to increase Δ*V_p-p_* and to decrease *n*:

1. To increase Δ*V_p-p_* by increasing the path length *L* is a straightforward way, but *L* is usually restricted by the full size of a gas detector, so this method can only contribute a small part.
2. Δ*V_p-p_* can also be increased by increasing *I_0_*(*λ_abs_*). One way to increase *I_0_*(*λ_abs_*) is to choose an IR source that gives off a stronger light intensity near *λ_abs_*, but this usually leads to a higher power consumption of the IR source and is not appropriate for a hand-held device; another way is to make full use of the intensity from the IR source, for example, by gilding the gas cell, by focusing the light beam onto the detection window of the IR detector.
3. Many different methods may be utilized for the reduction of the noise amplitude *n*. Traditional methods include hardware-based filters such as a Butterworth filter circuit, and software-based digital filters such as an moving average digital filter. However, these methods are not effective enough in pursuing a ppm level methane detection limit, and they may not be the best fits in the NDIR application. Recently Ye *et al.* reported that application of least-square fast transverse filtering (LS-FTF) to their NDIR methane sensor has greatly reduced the noise level such that an 8 ppm minimum detection limit has been achieved [12]. Here in this paper, we propose a single frequency filter algorithm based on Fourier transform, and the testing result of our methane sensor with this digital filter suggests that roughly a 1 ppm detection limit has been obtained.

### 2.2. Single Frequency Filter

The signal from the IR detector in an NDIR sensor is a sinusoidal or triangular-like wave with a dominating lamp modulation frequency. The signal may also contain components of interfering frequencies (for example the 50 Hz component from a commercial power supply) that should be eliminated in signal processing. Therefore, it should be beneficial to have a single frequency filter that allows only the lamp modulation frequency component to pass. We may use the amplitude of the discrete Fourier transform (DFT) at the lamp modulation frequency as the filtered signal, as shown in the following equation:

(6) $$F_{n}=\sum_{k=0}^{N-1}f_{k}e^{-2\pi \text{ink}/N}$$

where *f_k_* is the output signal of an IR detector, *N* is the number of total sampling points in one sampling period, *n* is the lamp modulation frequency, *F_n_* is the Fourier transform of the detector signal at lamp frequency. Moreover, *f_k_* can be written as:

(7) $$f_{k}=f_{ks}+f_{kn}$$

where *f_ks_* represents the signal component with the lamp modulation frequency, and *f_kn_* represents the noise of all possible frequencies. The filtered signal is then:

(8) $$\left|F_{n}\right|=\left|\sum_{k=0}^{N-1}(f_{ks}+f_{kn})e^{-2\pi \text{ink}/N}\right|$$

**Figure 2.**
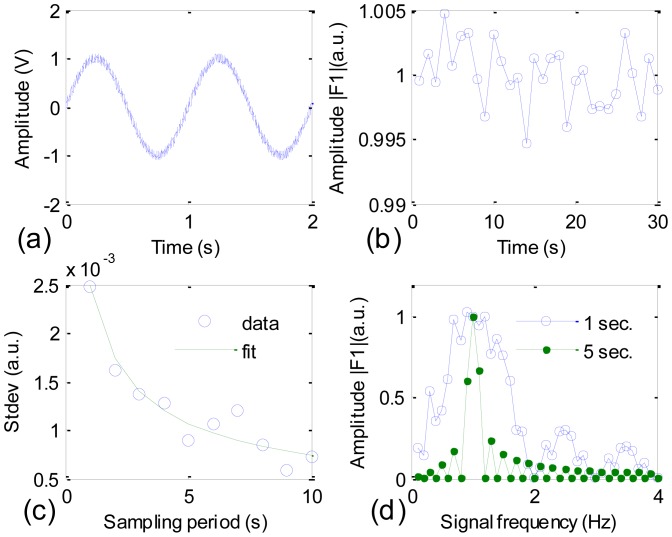
(**a**) Simulation of the signal of IR detector; (**b**) Normalized filter's output amplitude |*F_1_*| in arbitrary unit; (**c**) Sampling period dependence of the standard deviation of normalized amplitude |*F_1_*|; (**d**) Sampling period dependence of FWHM of the filter.

In order to evaluate the efficiency of this method, we did a simulation in LabView environment and some of the results are shown in Figure 2. Figure 2(a) shows a simulation IR detector's signal that is the superposition of a sine function of 1 V amplitude and 1 Hz frequency, and a uniform white noise of 0.1 V amplitude, or in terms of Equation (8)*f_ks_* = sin(2πt) and *f_kn_* = 0.1(white noise). Figure 2(b) shows the variation of the normalized amplitude |*F_1_*| (average of |*F_1_*| equals to 1) with the sampling period set at 1 s. The calculated standard deviation of |*F_1_*| is roughly 2.5 × 10^−3^, which is a good sign of noise reduction. The standard deviation becomes smaller as the sampling period increases, as shown in Figure 2(c).

The data points are fitted well with a t^−0.45^ function, which suggests that the effect of suppressing the noise level by extending the sampling period is most effective for the first few seconds. In Figure 2(d), |*F_1_*| is calculated with a 1 s sampling period (blue curve) and a 5 s sampling period (green curve). For each sampling period, the simulation signal is a pure sine function, in other words, *f_kn_* = 0; and *f_ks_* takes fractional frequencies, namely, *f_ks_* = sin(2πft) with *f* = 0.1, 0.2, …, 4. This is equal to say that sine functions with the same amplitude and different frequencies are filtered with the 1 Hz frequency filter. A comparison between the blue and green curves suggests that the FWHM of the single frequency filter is adjustable and the longer the sampling period, the narrower the FWHM should be. The result in Figure 2(d) explains from the spectrum point of view the tendency shown in the curve in Figure 2(c).

## 3. Experimental Section

**Figure 3.**
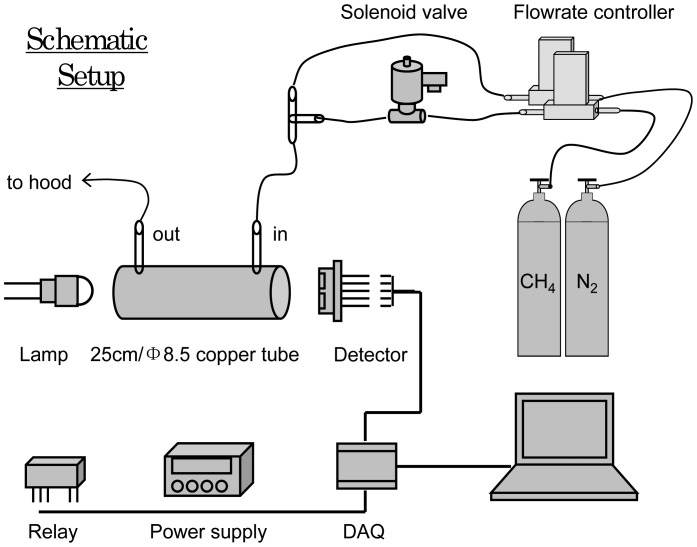
Schematic setup of the sensor prototype testing system.

The schematic setup of our sensor prototype and its testing system is shown in Figure 3. The IR source is an Mgg lamp (6004-10) that has a hemispherical lens on the top to facilitate the collimation of light. The homemade gas cell is a 25 cm long copper tube with a 8.5 mm inner diameter, the inner surface of the tube is gilded, and a 3 mm hole is drilled about 1.5 cm away from both ends of the tube as the gas inlet and outlet. The IR light detector is a pyroelectric methane detector from InfraTec (LIM-262), which is integrated with a NBP filter (3.3 μm/160 nm) and has a high detectivity and temperature stability. The detector's signal is collected by a data acquisition card (DAQ NI PCI-6225) and sent to a computer for data processing with a virtual instrument (VI). The computer sends a voltage square wave signal to a relay (TX2-5/ATX209) to modulate the IR lamp frequency and duty cycle. A DC power supply is used as the power source for the IR lamp and the IR detector.

The preparation procedure of a low ppm concentration of methane is as follows: the gas sources are a bottle of 50 ppm methane (CH_4_) and a bottle of pure nitrogen (N_2_). The gas flow from each bottle is controlled with a computer-controlled flow rate controller. After the controllers, the CH_4_ gas tube is connected to a solenoid valve, which has two positions to allow the gas either flow forward for gas mixing or to flow upward to be discarded. Then N_2_ gas and CH_4_ gas are mixed with a tee, and enter the gas cell from one end; the gas mixture leaves the cell from the other end and is discarded in a fume hood. Different CH_4_ concentrations are obtained by adjusting the flow rates of the two gas flows. For example, the mixture of 270 mL/min N_2_ and 30 mL/min CH_4_ (50 ppm) results in a CH_4_ concentration of 5 ppm. The gas flowrate from each flowrate controller is checked with an accurate portable rotameter before and after the measurement of every CH_4_ concentration to check the consistency of gas concentrations during the experiments.

**Figure 4.**
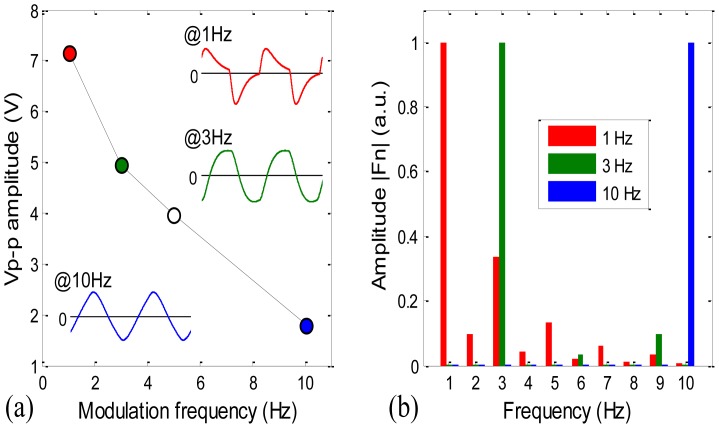
(**a**) Modulation depth and waveforms at different frequencies; (**b**) Spectra of different waveforms with a 1 s sampling period.

Figure 4(a) shows the modulation depth of the sensor with the lamp duty cycle set at 50%. The peak to peak amplitude *V_p-p_* of the IR detector's signal output decreases abruptly with the lamp modulation frequency from 1 to 10 Hz. Also shown in Figure 4(a) are the signal waveforms at 1, 3 and 10 Hz. The signal waveform changes apparently with the frequency. At 1 Hz, the waveform shows the features of a fast change away from 0 V and a slow recovery back to 0 V; these features become undistinguishable at 3 Hz, and at 10 Hz, the waveform becomes pretty much a triangular shape.

In order to evaluate the effect of the lamp modulation frequency on our filter's performance, we then compared the spectra of the waveforms at those three frequencies, shown in Figure 4(b). The sampling period is kept at 1 s for all three waveforms, the spectra are calculated with MATLAB function fft, and all three spectra are normalized such that the maximum amplitudes of them are equal to 1.

In Figure 4(b), the spectrum of the 1 Hz waveform has a second high amplitude of >0.3 at 3 Hz and some noticeable amplitudes at most other frequencies; the spectrum of the 3 Hz waveform has a second high amplitude of ∼0.1 at 9 Hz; and the spectrum of the 10 Hz waveform has a second high amplitude of about 1/9 at 30 Hz (not shown here). Moreover at 10 Hz, comparing with the one waveform (0.1 s sampling period) Fourier spectrum, the 10-waveform (1 s sampling period) Fourier spectrum shows a much narrow FWHM at the 10 Hz amplitude (result not shown here). This variation of spectrum suggests that the higher the lamp modulation frequency, the narrower the filter's band width (FWHM) will be, and therefore the smaller the noise level should be.

In summary, Figure 4(a) suggests that the signal strength is high at low frequencies; Figure 4(b) suggests that the noise level should be low at high frequencies with the digital filter based on Fourier transform. We expect that the combination of the two tendencies results in that at an intermediate frequency the system's SNR reaches a maximum, and consequently the system's detection limit reaches a minimum.

We then compiled a VI program for the sensor testing system in Figure 3. The main function of the VI is the calculation of the amplitude |*F_n_*| by FFT. The FFT mainly has two calculation steps with the Danielson-Lanczos lemma [19]. The first step sorts the data into bit-reversed order, *i.e.*, the original data array is sorted from the order j to its binary bit-reversed order j_r_ (for example, j:001 ≥ j_r_:100); the second step has a loop that is executed log_2_ *N* times and calculates, in turn, Fourier transforms of length 2, 4, 8, …, N.

**Figure 5.**
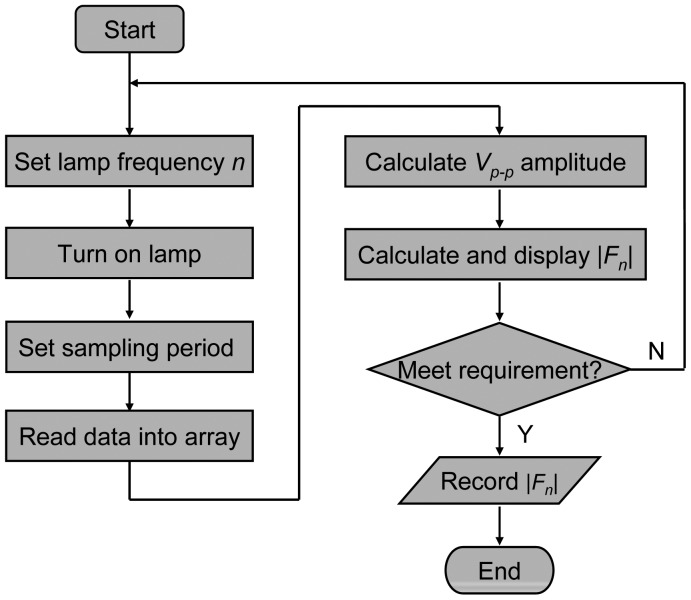
Main testing procedure of the methane sensor.

The sensor testing procedure in Figure 5 is described briefly as follows: first, the lamp modulation frequency *n* is set, the lamp is powered on, and the sampling period is set. Then, the computer reads the data into a data array, *V_p-p_* (indicator of signal strength) is calculated, and Fourier transform amplitude |*F_n_*| is calculated too. After that, the |*F_n_*| is displayed on computer when the gas flow is switched between CH_4_ and N_2_. If the variation of |*F_n_*| is satisfactory, then the data is saved to an output file and the program is stopped; otherwise, the program returns to the beginning, resets the lamp modulation frequency and sampling period, and attempts for a better detection effect. In addition, the switching between CH_4_ and N_2_ gases is done manually by means of the solenoid valve.

## 4. Results and Discussion

**Figure 6.**
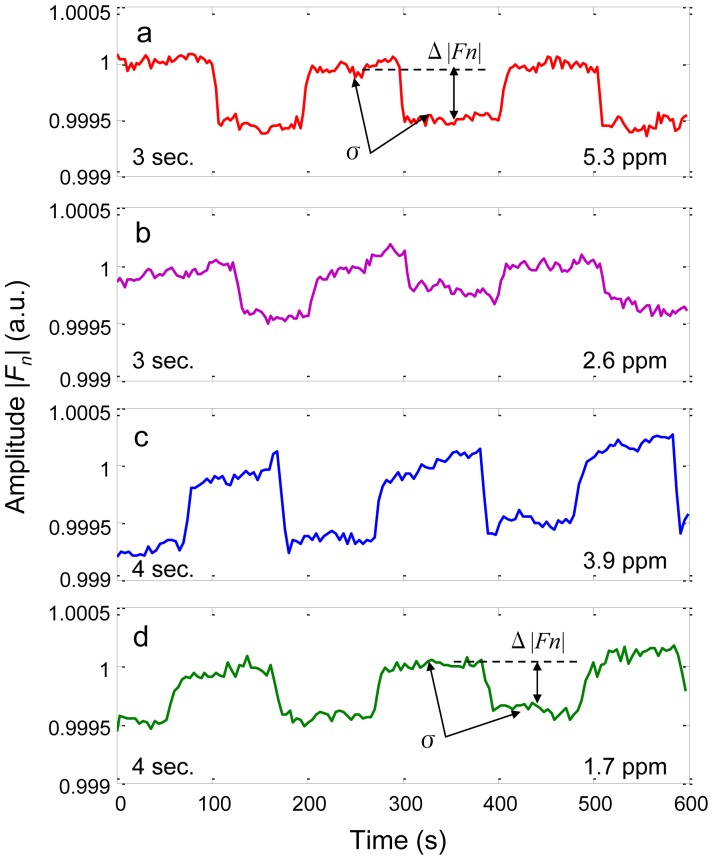
Detection effect on a 5.3 ppm methane (**a**) and a 2.6 ppm methane (**b**) with a 3 s sampling period. Detection effect on a 3.9 ppm methane (**c**) and a 1.7 ppm methane (**d**) with a 4 s sampling period.

The testing procedure in Figure 5 was performed repeatedly in an effort to narrow down the optimum lamp modulation frequency and sampling period that lead to the minimum detection limit. We found that the optimum frequency seemed to be around 3 Hz, and all the data in Figure 6 were taken at 3 Hz lamp modulation frequency. In Figure 6(a), the detection effect on a 5.3 ppm methane with a 3 s sampling period is quite stable. We may take the center square wave to estimate the detection limit. The signal strength is about Δ|*F_n_*| = 4.6E–4, and the standard deviation is about σ = 4.0E–5. If the detection limit is reached when the signal amplitude is three times of that of the standard deviation, the detection limit is estimated to be 1.4 ppm from this measurement.

We next doubled the N_2_ gas flowrate roughly and prepared a 2.6 ppm methane. Unfortunately, the testing result showed a much bigger fluctuation than anticipated, shown in Figure 6(b). We estimated that the abrupt degrading of the signal at 2.6 ppm may largely due to the high flow rate (close to 600 mL/min) in this measurement.

We then made some adjustments on the testing conditions to see if we could obtain an even lower detection limit. First, we managed to keep the total flowrate (N_2_ + CH_4_) low (<500 mL/min) by adjusting the CH_4_ flow rate to the possible minimum with the flow rate controller; second, we extended the sampling period from 3 s to 4 s; and third, we increased the lamp power supply from 5 V (rated value) to 5.5 V. With these modifications, the detection effect became apparently better.

Figure 6(c) shows the result on a 3.9 ppm methane sample. The signal clearly suffers from a DC drift that is mainly due to an insufficient warm up of the sensor system. Nevertheless, the signal strength is apparently greater than that in Figure 6(a) of the 5.3 ppm case. In Figure 6(d), the detection effect on a 1.7 ppm methane is more stable when the system was warmed up well. With the same estimation method discussed above with Figure 6(a), the detection limit from Figure 6(d) is then 0.42 ppm. We therefore conclude that the sensor system has the capability to detect a sub ppm level methane, yet the detection effect may be greatly deteriorated by the testing condition, such as insufficient warm up, or too big a gas flow rate.

The single digit ppm CH_4_ gas detection results in Figure 6 are unexpectedly good. We may pause and reflect on the key innovation of the sensor system, the single frequency filter. Fourier transform is well known, it is the selection of lamp modulation frequency to maximize the NDIR sensors' SNR makes it interesting. First, from Figure 4, increase of the modulation frequency makes the FWHM of the filter narrow and thus the noise level low, meanwhile, the light intensity from lamp decreases with the frequency and hence the signal strength decreases, the combination of these two effects leads to an optimum frequency for SNR; second, the optimum frequency is probably not an integer, we may consult the fractional Fourier transform algorithm [20] to construct a single fractional frequency filter to further improve the filter's performance. Judging from the abrupt decrease of signal strength in the modulation depth curve in Figure 4(a), this effort is probably worthy of future investigation; finally, in the calculation of our filter a rectangular window is used. It is well known that different windows have different merits in spectral analysis with DFT [21], it may also be worthwhile to examine the noise reduction effects with different windows in NDIR sensor's signal processing in the future.

A comparison between our methane sensor and some low ppm methane sensors is provided in Table 1. The size of our gas cell is larger than those of other sensors, however, our gas cell is low-cost and easy to manufacture. Owing mainly to the single frequency digital filter, our sensor reaches a 1 ppm methane detection limit, which is the lowest compared to that of others [12–15].

## 5. Conclusions

This paper describes the design and performance of an NDIR sensor system that is capable of detecting 1 ppm levels of methane gas. The design of the sensor aims at the enhancement of the system's SNR and mainly involves two measures: one, in order to increase the signal strength, a long (25 cm) cupper tube is chosen as the gas cell. Since the IR light is utilized efficiently by gilding the inner surface of the gas cell and by choosing the IR lamp with a hemispherical lens at top, the power consumption of the IR lamp is still compatible with a hand-held device.

The other and key measure is the single frequency digital filter based on FFT that greatly reduces the noise level. The performance of the filter is affected by the sampling period and the lamp modulation frequency. The longer the sampling period, the narrower the filter's FWHM will be, and hence the greater the system's SNR will be, provided that the signal is considerably stable over time. The determination of the lamp modulation frequency involves a two-fold consideration: (1) at low frequencies, the IR light intensity is high and hence the signal strength is high; (2) at high frequencies, the filter's FWHM is narrow and hence the noise level is low. The combination of the two factors implies that there should be an optimum frequency at which the system's SNR is the highest.

The sensor's detection effect is apparently influenced by the testing conditions, *i.e.*, the too big a gas flow rate or the insufficient warm up of the system. The former can be fixed by using a lower ppm (say 5 ppm) bottle methane to prepare the 1 ppm level gas; the latter may be improved by a better insulation of the lamp from the gas cell mechanically. Nevertheless, the detection effect on the 1.7 ppm methane suggests a sub ppm detection limit may be achievable, and we claim that a 1 ppm detection limit is achieved with our sensor system. This limit, to our knowledge, is much lower than that in the previous reports of methane sensors based on the NDIR principle.

## Figures and Tables

**Table 1.** Comparison between our methane sensor and some other low ppm methane sensors.

**Ref.**	**IR source**	**IR detector**	**Gas cell**	**Path length**	**Digital filter**	**Detection limit**
[14]	LED		HC1300 fiber	5.6 m		98 ppm
[15]	tunable laser		HC1600 fiber	5.1 m		10 ppm
[13]	LED	PbSe	ellipsoid	>4 cm		50 ppm
[12]	incandescent	thermopile	ellipsoid	>7.5 cm	LS-FTF	8 ppm
**this**	incandescent	pyroelectric	straight tube	25 cm	single-frequency	1 ppm
